# Preclinical antitumour activity of F 11782, a novel dual catalytic inhibitor of topoisomerases

**DOI:** 10.1054/bjoc.2000.1428

**Published:** 2000-11-22

**Authors:** A Kruczynski, C Etiévant, D Perrin, T Imbert, F Colpaert, B T Hill

**Affiliations:** Division of Experimental Cancer Research, Division of Medicinal Chemistry III, Research Centre Directorate, Centre de Recherche Pierre Fabre, 17 avenue Jean Moulin, Castres, Cedex 06, 81106, France

**Keywords:** in vivo cancer chemotherapy, dual topoisomerase inhibitor

## Abstract

F 11782 is a novel inhibitor of topoisomerases I and II, with an original mechanism of action (Perrin et al, 2000). This study, aimed to define its anticancer efficacy against a series of murine and human tumour models, has provided evidence of major antitumour activity for F 11782. This was demonstrated as a high level of activity against the P388 leukaemia, as reflected by increased survival of 143–457%, when administered i.p., p.o. or i.v. as single or multiple doses, and proved consistently superior to etoposide or camptothecin tested concurrently. Single or multiple i.p. doses of F 11782 also proved highly active against the s.c. grafted B16 melanoma, significantly increasing survival (P < 0.001) and inhibiting tumour growth (T/C of 0.3%), again superior to etoposide tested concurrently. Furthermore, F 11782 inhibited the number of pulmonary metastatic foci of the B16F10 melanoma by 99%. In human tumour xenograft studies, multiple i.p. doses of F 11782 resulted in major inhibitory activity against MX-1 (breast) tumours (T/C of 0.1%), as well as causing definite tumour regressions, whereas none resulted from similar experimental treatments with etoposide. Significant activity was also recorded with F 11782 against the relatively refractory LX-1 (lung) xenografts, with an optimal T/C value of 19%. It was notable that the antitumour activity of F 11782 was consistently demonstrated over a wide range of 2–6 dose levels, providing evidence of its good overall tolerance. In conclusion, these results emphasize the preclinical interest of this novel molecule and support its further preclinical development. © 2000 Cancer Research Campaign http://www.bjcancer.com


British Journal of Cancer (2000) 83(11), 1516–1524
© 2000 Cancer Research Campaign
doi: 10.1054/ bjoc.2000.1428, available online at http://www.idealibrary.com on IDEAL®

http://www.bjcancer.com

### Preclinical antitumour activity of F 11782, a novel dual catalytic inhibitor of topoisomerases

**A Kruczynski[1,4], C Etiévant[1], D Perrin[1], T Imbert[2], F Colpaert[3] and B T Hill[1]**

[1]Division of Experimental Cancer Research, [2]Division of Medicinal Chemistry III and [3]Research Centre Directorate, Centre de Recherche Pierre Fabre, 17 avenue Jean Moulin, 81106 Castres, Cedex 06, France

Summary F 11782 is a novel inhibitor of topoisomerases I and II, with an original mechanism of action (Perrin et al, 2000). This study, aimed to define its anticancer efficacy against a series of murine and human tumour models, has provided evidence of major antitumour activity for F 11782. This was demonstrated as a high level of activity against the P388 leukaemia, as reflected by increased survival of 143–457%, when administered i.p., p.o. or i.v. as single or multiple doses, and proved consistently superior to etoposide or camptothecin tested concurrently. Single or multiple i.p. doses of F 11782 also proved highly active against the s.c. grafted B16 melanoma, significantly increasing survival ($P < 0.001$) and inhibiting tumour growth (T/C of 0.3%), again superior to etoposide tested concurrently. Furthermore, F 11782 inhibited the number of pulmonary metastatic foci of the B16F10 melanoma by 99%. In human tumour xenograft studies, multiple i.p. doses of F 11782 resulted in major inhibitory activity against MX-1 (breast) tumours (T/C of 0.1%), as well as causing definite tumour regressions, whereas none resulted from similar experimental treatments with etoposide. Significant activity was also recorded with F 11782 against the relatively refractory LX-1 (lung) xenografts, with an optimal T/C value of 19%. It was notable that the antitumour activity of F 11782 was consistently demonstrated over a wide range of 2–6 dose levels, providing evidence of its good overall tolerance. In conclusion, these results emphasize the preclinical interest of this novel molecule and support its further preclinical development. © 2000 Cancer Research Campaign http://www.bjcancer.com

**Keywords:** in vivo cancer chemotherapy; dual topoisomerase inhibitor

A wide range of topoisomerase-targeted agents are useful clinically, with documented activities in a broad spectrum of human malignancies (Pommier, 1997). Most of these have been shown to stabilize the reaction intermediate of the topoisomerase catalytic cycle, termed the cleavable complex, resulting in DNA cleavage stimulation and this is considered the molecular basis for their antitumour activities (Froelich-Ammon and Osheroff, 1995; Pommier, 1997). These types of agents have been termed topoisomerase poisons (Gatto et al, 1999). However, other compounds have more recently been shown to interfere with topoisomerase function without stabilizing the cleavable complex and these, termed catalytic inhibitors, include merbarone (Khélifa and Beck, 1999), bis(dioxopiperazine) derivatives (Ishida et al, 1995) and certain DNA-interacting agents (Gatto et al, 1999).

Amongst various topoisomerase poisons, a few have been reported to possess dual inhibitory activities against both topoisomerases I and II, including saintopin (Yamashita et al, 1991), intoplicine (Riou et al, 1991) and TAS-103 (Fortune et al, 2000). All these dual inhibitors though share DNA intercalating properties and stabilize cleavable complexes. Indeed, inhibition of the function of one topoisomerase is frequently compensated for by alterations in the expression of the other. Furthermore, expression levels of topoisomerases I and II vary with tumour type (van der Zee et al, 1991; Hussain et al, 1994). Therefore, targeting both topoisomerases appears an attractive chemotherapeutic strategy and in aiming to identify new antitumour compounds with broader activity spectra, a catalytic inhibitor of both these nuclear enzymes was considered a potential candidate.

Thus, a research programme was initiated centred on synthesizing chemical modifications of epipodophyllotoxin-based compounds, using the etoposide skeleton, considering that enhanced lipophilicity would be associated with increased interaction with lipidic cell membranes and improved cellular penetration. It was hypothesized that such enhanced lipophilicity would promote an important modification in the compound's in vivo distribution, so leading to new epipodophylloid-type compounds with a novel profile of antitumour activity. F 11782 or 2″,3″,-bis pentafluorophenoxyacetyl-4″,6″-ethylidene-β-D glucoside of 4′-phosphate-4′-demethylepipodophyllotoxin, N-methyl glucamine salt was selected from this series based on its activity in primary pharmacological screening and its original mechanistic properties as a dual catalytic inhibitor of both topoisomerases I and II without DNA intercalating properties (Perrin et al, 2000).

This report focuses on the in vivo pharmacological profile of F 11782 against a panel of transplantable tumour models with different biological properties and chemosensitivities, specifically the i.v.-implanted murine P388 leukaemia, the s.c.-implanted B16 and the metastatic i.v.-implanted B16F10 melanoma, and two human tumour xenografts MX-1 (breast) and LX-1 (lung).

*Received 29 March 2000*
*Revised 21 June 2000*
*Accepted 23 June 2000*

*Correspondence to:* A Kruczynski

### MATERIALS AND METHODS

### Drugs

F 11782 (CRPF, Castres, France) was dissolved in a solution containing 5% Tween in 5% glucose in water (5/95; w/v) for i.p.

and p.o. administrations, and in 100% DMSO for i.v. injections. Etoposide (Pierre Fabre Médicament, Castres, France) and camptothecin (Janssen, Noisy Le Grand, France) were solubilized in a 0.9% sodium chloride containing 5% Tween (v/w).

### Mice and tumour models

Female hybrid CDF1 (CD2F1/Cr1BR) and C57BL/6 (C57BL/6 NCr1BR) mice (Charles River, St Aubin-les-Elbeuf, France) were used for implanting the murine P388 leukaemia, and the murine B16 and B16F10 melanomas (Division of Cancer Treatment, Tumour Repository, NCI, Frederick, MD, USA), respectively. Homozygous female athymic nude mice (Ico: Swiss-nu/nu, Iffa Credo, L'Arbresle, France), were implanted with LX-1 (lung) or MX-1 (breast) human tumour xenografts (Division of Cancer Treatment, Tumour Repository, NCI, Frederick, MD, USA). Animals were handled and cared for in accordance with the Guide for the Care and Use of Laboratory Animals (National Research Council, 1996) and the European Directive EEC/86/609, under the supervision of authorized investigators.

### Experimental chemotherapy

All experiments were conducted in compliance with French regulations and CRPF ethical committee guidelines, based on the UKCCCR guidelines for the welfare of animals in experimental neoplasia, as detailed previously (Kruczynski et al, 1998). $10^6$ P388 cells/mouse were implanted i.v. into C2DF1 mice on day zero. For the B16 model, 0.5 ml of a tumour brei at 1 g/ml, made by disrupting and homogenizing tumour fragments in sterile 0.9% sodium chloride were inoculated s.c. into mice on day zero. Fragments of human tumour xenografts were implanted into Swiss mice by trocar and allowed to increase to a median value of 100–300 and 46–150 $mm^3$ for the advanced-stage and early-stage disease, respectively. After randomization in treatment cages, test compounds were administered according to various schedules and/or routes. B16F10 cells maintained in vitro in RPMI medium supplemented with 10% fetal calf serum, 4 mM L-glutamine, 1.25 μg/ml fungizone and 100 μg/ml penicillin-streptomycin, were implanted i.v. into the tail vein of mice, and test compounds were administered the following day. In each chemotherapy trial, mice were checked daily, with any adverse clinical reactions noted and deaths recorded. Mice were weighed 2–4 times weekly during treatments and once weekly thereafter. Tumours were measured by callipers twice weekly and tumour volumes ($mm^3$) were estimated as = 0.5 (length × width$^2$). Results are presented for experiments involving 7–30 mice per experimental group according to the model used.

### Evaluations of antitumour activity

#### *Life span*

An increase of survival was defined as the (median survival of treated mice/median survival of control mice) × 100, ($T/C_{ls}$ %). Survival curves of treated and control groups of B16 tumour-bearing mice were statistically compared using the Log-rank test.

#### *Tumour growth*

Treatment efficacy was assessed in terms of the compound's effects on the tumour volumes of tumour-bearing relative to control vehicle-treated mice. Four evaluation criteria were used in parallel: (i) Growth inhibition, calculated as the ratio of the median tumour volumes of drug-treated versus control groups: T/C, % = (median tumour volume of drug-treated group on day X /median tumour volume of control group on day X) × 100, the optimal value, being the minimal T/C ratio which reflects the maximal tumour growth inhibition achieved (Hendriks et al, 1992); (ii) Specific tumour growth delay (SGD), calculated as follows: for the B16 model = [Td (drug-treated group) – Td (vehicle-treated group)]/Td (vehicle-treated group), with Td being the tumour doubling time of drug-treated and control groups, defined as the time in days required for the tumour volume to double. For human tumour xenografts, SGD was defined as the difference in time for drug-treated and control tumours to reach a given volume, (v), divided by the time for the control tumours to reach this same volume v. Thus, $SGD_v = [T_v$ (drug-treated group) – $T_v$ (control group)]/$T_v$ (control group)], with the v value being 1500 $mm^3$ and 500 $mm^3$ for the MX-1 and LX-1 tumour xenografts, respectively, and $T_v$ being the time for drug-treated or control groups to reach the given volume v; (iii) Tumour regressions, defined as partial (PR) if the tumour volume decreased to 50% or less of that at the start of treatment, without dropping below measurable size (Plowman et al, 1997); (iv) Relative area under the tumour growth curve, rAUC (%), representative of the tumour growth curve as a whole, reflects the overall effect of a test compound over time (cf. Kruczynski et al, 1998). rAUC = median [(area under the tumour growth curve of an individual experimental mouse/median area under the tumour growth curve of the control group) × 100]. The more active the compound, the lower the rAUC value. The non-parametric Mann-Whitney Rank Sum test was used for statistical comparisons of the respective rAUC population values.

#### *Lung metastatic foci*

On day 17 after tumour cell implantation, lungs were quickly excised, the metastatic foci on the pulmonary surface were counted. Results of control and drug-treated groups are expressed as median values.

### RESULTS

### P388 murine leukaemia

F 11782 demonstrated marked antitumour activity against the i.v.-implanted P388 leukaemia, given i.p. as a single dose. The optimal dose, i.e., that inducing the greatest increase of life span, reflected by a maximal $T/C_{ls}$ ratio with minimal side effects, was 320 mg/-kg, producing a median $T/C_{ls}$ value of 400%, considered indicative of a high level of activity (H) by NCI criteria (Table 1), with body weight loss of only 5.6%. Doses of F 11782 resulting in definite toxicity were not identified, since those higher than 320 mg/kg could not be evaluated because of limited solubility and the high viscosity of F 11782-containing solutions. The reference compounds studied, etoposide and camptothecin, were also active against this i.v.-implanted P388 leukaemia, but gave lower optimal $T/C_{ls}$ values of only 214–243%.

The effects of multiple doses of F 11782 (Table 1), either as four daily injections (days 1–4), or as intermittent treatments over 2 weeks (days 3, 5, 7, 10, 12 and 14), also provided evidence of a high level of activity with an increased life span reflected by maximal $T/C_{ls}$ ratios of 429% and 457%. Using the 2-week

© 2000 Cancer Research Campaign

**Table 1** Antitumour activity of F 11782 given i.p. as a single or multiple doses against the i.v.-implanted P388 murine leukaemia. Comparison with etoposide and camptothecin

| Test compound | Schedule (days) | Dose (mg/kg) Single | Total | Max. body weight change[a] (%) [day] | Presumed drug-related deaths[b] (%) | T/C[c](%) | Activity rating[d] |
|---|---|---|---|---|---|---|---|
| F 11782 | ‘single dose’ | 40 | 40 | –0.7 [4] | 0 | 143 | L |
| | (1) | 80 | 80 | gain | 0 | 200 | H |
| | | 160 | 160 | –1.9 [4] | 0 | 300 | H |
| | | 320# | 320# | –5.6 [8] | 0 | 400 | H |
| etoposide | ‘single dose’ | 20 | 20 | gain | 0 | 157 | L |
| | (1) | 80 | 80 | –2.3 [4] | 0 | 214 | H |
| | | 160 | 160 | –6.1 [4] | 0 | 243 | H |
| | | 640 | 640 | –11 [4] | 86 | 86 | toxic |
| camptothecin | ‘single dose’ | 5 | 5 | 0.0 [4] | 0 | 157 | L |
| | (1) | 10 | 10 | –4.9 [4] | 0 | 200 | H |
| | | 20 | 20 | –9.8 [4] | 0 | 214 | H |
| | | 40 | 40 | –13.0 [4] | 43 | 100 | toxic |
| F 11782 | ‘1 week’ | 5 | 20 | –0.9 [3] | 0 | 143 | L |
| | 1,2,3,4 | 20 | 80 | –1.7 [2] | 0 | 229 | H |
| | | 40 | 160 | –1.8 [3] | 0 | 286 | H |
| | | 80 | 320 | –3.0 [2] | 0 | 421 | H |
| | | 160 | 640 | –11.7 [8] | 0 | 429 | H |
| | | 320# | 1280 | –15.2 [4] | 14 | 86 | 0 |
| F 11782 | “2 weeks” | 10 | 60 | –1.8 [12] | 0 | 157 | L |
| | 3,5,7,10,12,14 | 20 | 120 | gain | 0 | 257 | H |
| | | 40 | 240 | gain | 0 | 257 | H |
| | | 80 | 480 | –2.7 [5] | 0 | 286 | H |
| | | 160 | 960 | –7.5 [14] | 0 | 457 | H |
| | | 320[e] | 1920 | –28.9 [10] | 0 | 143 | L |

[a]Body weight changes are maximal weight losses expressed as a percentage of initial body weight. According to NCI criteria, a dose is considered toxic if the induced body weight loss is >15% of the initial body weight. No body weight loss was recorded in control animals. [b]A death was presumed drug-related if it preceded the first death in the solvent-treated group. [c]T/C = (median survival of drug-treated group/median survival of control group) × 100. [d]According to NCI standard criteria for the P388 tumour model, 120% ≤ T/C < 175% is the minimal level for activity (L) and T/C ≥ 175% corresponds to a high level of antileukaemic activity (H); “0” represents a T/C value of < 120% (Venditti, 1981). [e]Higher doses of F 11782 were not tested because of its limited solubility and high viscosity in solution. Max. = maximal schedule, multiple doses of up to 160 mg/kg/injection induced no major body weight loss (Table 1). However, four daily injections of 320 mg/kg F 11782 resulted in 14% early deaths, probably associated with solubility/viscosity problems, which appeared cumulative with repeated dosing, since at autopsy following the last treatment whitish deposits were noted within the peritoneal cavities of treated mice. Six treatments with 320 mg/kg/injection of F 11782 over two weeks also induced marked weight loss of 28.9% (Table 1) and again at autopsy whitish deposits were revealed within the peritoneal cavity.

F 11782 was also active against the i.v.-implanted P388 leukaemia, administered i.v. or p.o., in a single dose, inducing an increased life span reflected by maximal $T/C_{ls}$ ratios of 314% and 143%, respectively (Table 2). Indeed i.v. administration of 150 mg/kg F 11782 resulted in 3/7 (43%) long-term survivors at 60 days. No weight loss in excess of 9% was recorded, although one presumed drug-related death was recorded at 160 mg/kg (Table 2). The activity of F 11782 using the p.o. route, was greatly augmented when multiple intermittent treatments were given over 2 weeks, reflected by $T/C_{ls}$ values of 243% and these repeated doses were associated with body weight losses of only 1–2% (Table 2).

### B16 murine melanoma

Activity was evaluated using a single dose, multiple daily injections (days 3–6) or intermittent treatments over 2 weeks, i.e., on days 3, 5, 7, 10, 12 and 14 (Table 3). F 11782 given i.p. as a single dose significantly increased survival, as assessed by the Log-rank test ($P < 0.001$), and at 320 mg/kg was associated with significant tumour growth inhibition, reflected by an optimal T/C value of 3% judged, according to NCI criteria, as a high level of activity (T/C < 10%) and a significant ($P < 0.001$) rAUC values of 17% (Table 3). Significant tumour growth inhibition was also recorded at 160 mg/kg F 11782, with an optimal T/C value of 24% and an rAUC value of 27%. Again, doses of F 11782 higher than 320 mg/kg could not be tested because of limited solubility/high viscosity and therefore toxic doses were not identified. Multiple daily administrations (on days 3–6) significantly increased survival ($P < 0.001$–0.05) at doses of 20, 40 and 80 mg/kg/injection, associated with marked effects on tumour growth reflected by significant (≤ 42%) optimal T/C ratios of 29%, 4% and 0.3%, respectively and significant ($P < 0.001$) rAUC values of 39%, 26% and 17%, respectively (Table 3). Again the T/C values of 4% and 0.3% are representative of a high level of activity (T/C < 10%). No body weight loss in excess of 2.7% was recorded. However, at 160 mg/kg/injection 18% weight loss and some immediate toxic deaths resulted. Administration of F 11782 as intermittent treatments over 2 weeks had marked antitumour activity against this s.c.-implanted B16 melanoma, both in terms of increased life span ($P < 0.001$–0.01) and tumour growth inhibition. These effects were observed at four dose levels, ranging from 20–160 mg/kg (Table 3 and Figure 1), yielding optimal T/C ratios of 0.2–24%, with three doses providing a high level of antitumour activity (T/C

 *© 2000 Cancer Research Campaign*

**Table 2** Effects of the route of administration on the antitumour activity of F 11782 against the i.v.-implanted P388 leukaemia[a]

| Schedule (days) | Dose (mg/kg) Single | Total | Route | Maximal body weight change (%) [day] | Presumed drug-related deaths (%) | T/C (%) [Long-term survivors, 60 days][b] | Activity rating |
|---|---|---|---|---|---|---|---|
| 'single dose' | 40 | 40 | i.v. | gain | 0 | 114 | 0 |
| (1) | 80 | 80 | i.v. | gain | 0 | 186 | H |
| | 120 | 120 | i.v. | –1.5 [4] | 0 | 300 | H |
| | 150 | 150 | i.v. | –6.2 [8] | 0 | 314 [3/7] | H |
| | 160 | 160 | i.v. | –1.2 [4] | 14 | 257 [1/7] | H |
| 'single dose' | 40 | 40 | p.o. | gain | 0 | 114 | 0 |
| (1) | 80 | 80 | p.o. | gain | 0 | 129 | L |
| | 160 | 160 | p.o. | –1.0 [8] | 0 | 129 | L |
| | 320# | 320 | p.o. | gain | 0 | 143 | L |
| '2 weeks' | 2.5 | 15 | p.o. | gain | 0 | 100 | 0 |
| (3,5,7,10,12,14) | 10 | 60 | p.o. | –2.0 [10] | 0 | 143 | L |
| | 20 | 120 | p.o. | gain | 0 | 129 | L |
| | 40 | 240 | p.o. | –2.8 [10] | 0 | 143 | L |
| | 80 | 480 | p.o. | –9.0 [12] | 0 | 200 | H |
| | 160 | 960 | p.o. | –1.0 [12] | 0 | 243 | H |
| | 320# | 1920 | p.o. | –2.0 [14] | 0 | 243 | H |

[a]See footnotes to Table 1. [b]Treated-animals still surviving 60 days after tumour implantation are recorded as long-term survivors.

**Table 3** Antitumour activity of F 11782 given i.p. against the s.c.-implanted B16 murine melanoma: comparison with etoposide

| Test compound | Schedule (days) | Dose (mg/kg/inj) | Maximal body weight change[a] (%) [day] | Survival Log-rank test | Tumour growth inhibition Opt.T/C[b] (%) [day] | SGD[c] | rAUC[d] (%) [Mann-Whitney test] |
|---|---|---|---|---|---|---|---|
| F 11782 | 'single dose' | 80 | gain | ns | 70 [12] | 0.0 | 98 [ns] |
| | (3) | 160 | –2.5 [5] | ns | 24 [12] | 1.1 | 27 [***] |
| | | 320# | –4.7 [5] | *** | 3 [12] | 0.6 | 17 [***] |
| F 11782 | 'qld×4' | 10 | gain | ns | 83 [19] | <0 | 98 [ns] |
| | (3,4,5,6) | 20 | gain | * | 29 [12] | 0.3 | 39 [***] |
| | | 40 | gain | ** | 4 [12] | 0.3 | 26 [***] |
| | | 80 | –2.7 [10] | *** | 0.3 [12] | 0.7 | 17 [***] |
| | | 160 | –18 [10] | | | toxic deaths[e] | |
| F 11782 | '2 weeks' | 10 | gain | ns | 96 [17] | <0 | 120 [ns] |
| | (3,5,7,10,12,14) | 20 | gain | ** | 4 [21] | 4.0 | 30 [***] |
| | | 40 | gain | *** | 24 [17] | 3.3 | 43 [***] |
| | | 80 | –4.4 [14] | *** | 3 [21] | 6.6 | 4 [***] |
| | | 160 | –27 [21] | ** | 0.2 [21] | 2.8 | 1 [***] |
| etoposide | '2 weeks' | 10 | 0.0 [5] | * | 48 [14] | 0.5 | 68 [*] |
| | (3,5,7,10,12,14) | 20 | gain | ns | 42 [17] | 0.5 | 53 [**] |
| | | 40 | –2.7 [10] | ns | 13 [14] | 0.6 | 15 [***] |
| | | 80 | –22 [12] | | | toxic deaths | |

[a]Body weight changes are maximal weight losses expressed as a percentage of intial body weight. No body weight loss was recorded in control animals. [b]T/C = (median tumour volume of drug-treated group/median tumour volume of control group) × 100. According to NCI standard criteria for a solid tumour model, T/C ≤ 42% corresponds to a minimal level of activity (Bissery et al, 1991). [c]SGD = [Td (drug-treated group) – Td (control group)]/Td control group, with Td being the time required for the tumour to double in volume. According to NCI/EORTC standard criteria, SGD > 1 corresponds to a minimal level of activity (Langdon et al, 1994). [d]rAUC = relative area under the tumour growth curve. [e]Toxic deaths, i.e., when treated animals died before controls. ns = $P > 0.05$; * = $P < 0.05$;** = $P < 0.01$;*** = $P < 0.001$. #Higher doses of F 11782 were not tested because of its limited solubility and high viscosity in solution. Opt. = optimal

< 10%). This tumour growth inhibition was also illustrated by significant ($P < 0.001$) rAUC values of 1–43%, as well as markedly significant SGD values of 2.8–6.6 (Table 3), i.e., > 1 according to the criteria of Langdon et al (1994). However, the highest dose of 160 mg/kg/injection resulted in toxicity with body weight loss of 27%. Therefore, the optimal tolerated dose of F 11782 given over 2 weeks was 80 mg/kg/injection corresponding to an optimal total dose of 480 mg/kg, which was 1.5-fold higher than that which could be given either as a single-dose or as multiple daily treatments (Table 3). Therefore, intermittent treatments over 2 weeks enabled a higher optimal total dose of F 11782 to be administered and resulted in the highest level of antitumour activity against this s.c.-implanted B16 model.

A comparative study conducted in parallel showed that etoposide, given as intermittent treatments over 2 weeks, induced only minimal inhibitory activity (T/C of 13%) and an rAUC value of 15% at 40 mg/kg/injection (Table 3). Increasing the dose to 80 mg/kg/injection resulted in toxicity with body weight loss of 22%, as well as toxic deaths, not though associated with any solubilization problems (Table 3). Administration of etoposide as

© 2000 Cancer Research Campaign

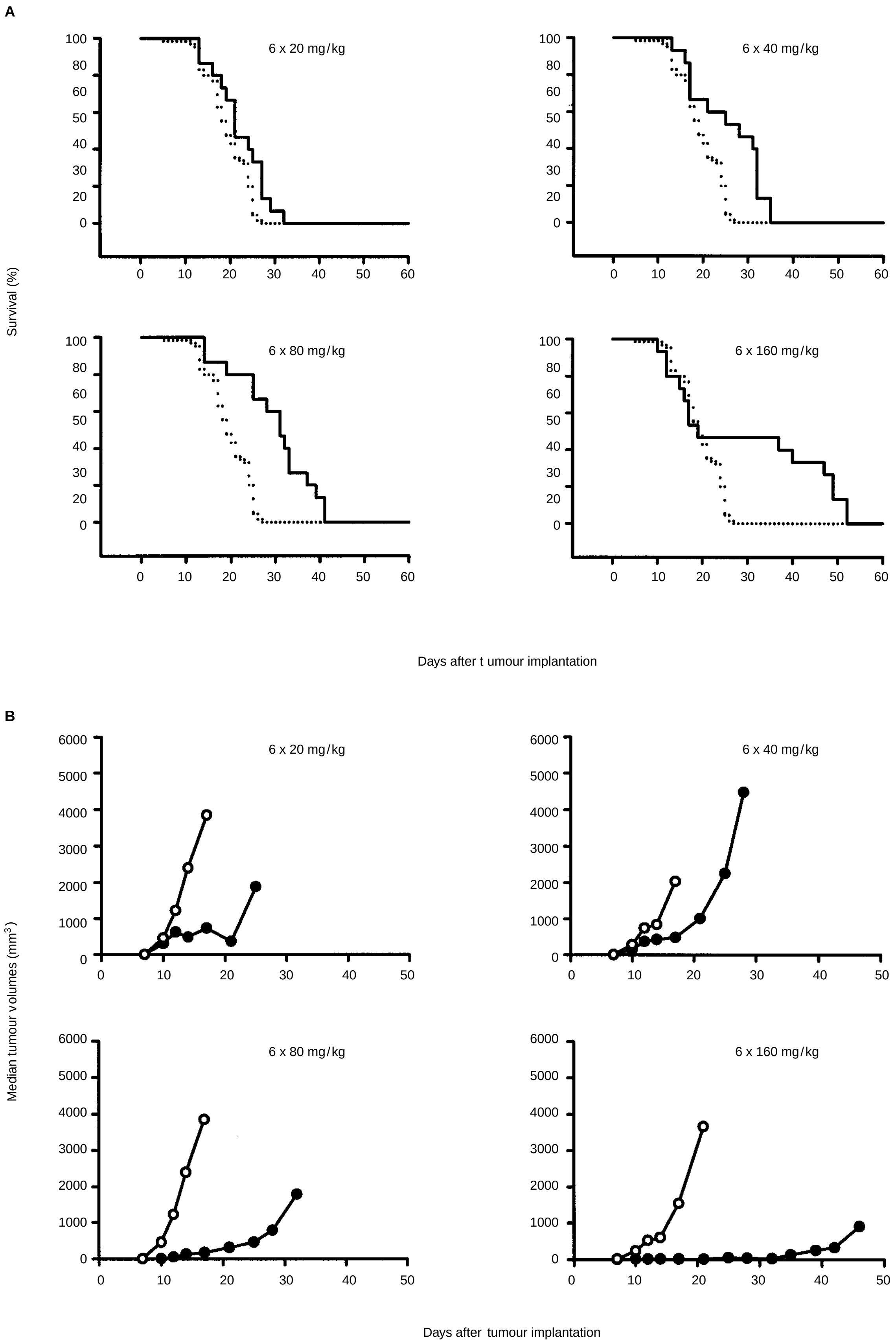

**Figure 1** Effects of F 11782 given i.p. as intermittent treatments over two weeks (days 3, 5, 7, 10, 12 and 14) at 20, 40, 80 or 160 mg/kg per injection on the survival (**A**) and tumour growth (**B**) of s.c.-grafted B16 melanoma-bearing mice. Initially, 0.5 ml of B16 tumour brei at 1 mg/ml were inoculated s.c. into C57BL/6 mice, and drug treatments were initiated 3 days later. The solid and dotted lines correspond to the F 11782-treated and vehicle-treated groups, respectively

*© 2000 Cancer Research Campaign*

**Table 4** Antitumour activity of F 11782 given i.p. against the s.c.-implanted human MX-1 (breast) and LX-1 (lung) tumour xenografts: comparison with etoposide[a]

| Model | Test compound | Dose (mg/kg/inj) | Maximal body weight change (%) [day] | Tumour growth inhibition/regression | | |
|---|---|---|---|---|---|---|
| | | | | Opt.T/C (%) [day] | SGDv[b] [PR[c], duration in days] | rAUC (%) [Mann-Whitney test] |
| Advanced-stage | F 11782 | 40 | –2.1 [13] | 69 [26] | 0.2 | 113 [ns] |
| MX-1 | | 80 | –6.5 [13] | 17 [36] | 2.3 | 27 [***] |
| (Breast) | | 120 | –4.0 [13] | 0.8 [40] | [PR, 33–47] | 11 [***] |
| | | 160 | –3.4 [15] | 0.1 [36] | [PR, 29–47] | 11 [***] |
| | control | | gain | | | |
| | etoposide | 20 | gain | 77 [22] | 0.2 | 89 [ns] |
| | | 30 | –8.1 [18] | 44 [22] | 0.8 | 48 [**] |
| | | 40 | –21.3 [26] | 4 [29] | 2.3 | 22 [***] |
| | control | | gain | | | |
| Early-stage | F 11782 | 80 | –5.6 [9] | 56 [39] | 0.1 | 85 [ns] |
| LX-1 | | 120 | –6.8 [11] | 41 [36] | 0.4 | 53 [**] |
| (Lung) | | 160 | –11.3 [22] | 19 [39] | 1.9 | 29 [***] |
| | control | | –0.4 [11] | | | |
| | etoposide | 20 | –2.6 [36] | 45 [15] | 0.5 | 90 [ns] |
| | | 30 | –6.8 [14] | 35 [15] | 1.0 | 70 [*] |
| | | 40 | –16.2 [25] | 27 [15] | 2.0 | 45 [***] |
| | control | | –0.4 [25] | | | |

[a]See footnotes to Table 3. [b]SGDv = [Tv (drug-treated group) – Tv (control group)]/Tv (control group), with Tv being the time for treated and control tumours to reach a given volume v; v being 1500 $mm^3$ and 500 $mm^3$ for the MX-1 and LX-1 models, respectively. [c]PR = partial tumour regression. Test compounds were given i.p. as intermittent treatments over two weeks, on days 11, 13, 15, 18, 20, 22, and 7, 9, 11, 14, 16, 18 for the MX-1 and LX-1 tumour models, respectively. ns = $P$ >0.05; * = $P$ <0.05; ** = $P$ < 0.01; *** = $P$ <0.001.

single or multiple daily (days 3–6) doses also provided only minimal antitumour effects in terms of growth inhibition, as reflected by optimal T/C ratios of 41% and 20%, and optimal rAUC values of 66% and 40%, respectively (data not shown).

### Experimental B16F10 pulmonary metastases

F 11782 given i.p. in a single dose on the day following tumour implantation resulted in a marked dose-dependent reduction of pulmonary B16F10 metastatic foci number in the lungs of mice, with inhibitions of 66.5, 82.5, 91 and 99% noted at 40, 80, 160 and 320 mg/kg, respectively (data not shown). No body weight loss in excess of 15% was recorded.

### Human MX-1 (breast) and LX-1 (lung) tumour xenografts

F 11782 exhibited major activity against advanced-stage MX-1 human breast xenografts (Table 4). This activity was obtained following intermittent i.p. treatments over 2 weeks at doses of 80, 120 and 160 mg/kg/injection. A high level of antitumour activity resulted from the two highest doses, with definite tumour regressions, reflected by partial regressions lasting from days 33–47 and 29–47 post-tumour implantation, respectively. Optimal T/C ratios of < 10%, namely 0.8% and 0.1%, also indicated a high level of activity according to NCI criteria. This antitumour activity was sustained over time since T/C ratios < 10% were recorded from day 26 until the end of the experiments on day 47. Highly significant ($P$ < 0.001) rAUC measurements resulted from these two highest doses, as well as at the lower dose of 80 mg/kg/injection, which was associated with a significant (i.e., > 1) $SGD_{1500}$ value of 2.3. No body weight loss in excess of 6.5% was noted. Attempts to evaluate 240 mg/kg/injection produced early deaths (data not shown), most probably associated with the solubility/high viscosity problems, as discussed above.

F 11782 showed clearly superior activity to that of etoposide against similarly staged MX-1 tumour xenografts (see Figure 2 and Table 4). Etoposide exhibited only moderate activity, with high level of activity being recorded only at the highest dose tested of 40 mg/kg/injection, which though was toxic being associated with significant body weight loss of 21%.

The responses of LX-1 human lung xenografts to F 11782 given i.p. as intermittent treatments over 2 weeks are detailed in Table 4. Starting treatments when tumours reached a volume range of 46–150 $mm^3$, resulted in definite tumour growth inhibition at 120 and 160 mg/kg F 11782 and rAUC values for the treated animals significantly ($P$ < 0.001–0.01) lower than those of control mice (Table 4). Optimal T/C ratios of 41% and 19%, judged significant (i.e., ≤ 42%) were noted et 120 and 160 mg/kg/injection, respectively and at the higher dose the $SGD_{500}$ value of 1.9 was significant (i.e., > 1) and was associated with body weight loss of only 11.3% (Table 4).

Etoposide, evaluated concurrently, showed moderate activity against these LX-1 tumours, with significant effects recorded at the non-toxic dose of 30 mg/kg/injection, reflected by an rAUC value of 70% and an optimal T/C ratio of 35%. Increasing the dose, whilst resulting in improved antitumour activity, was associated with some toxicity involving body weight loss of 16.2% (Table 4). These results illustrate the superior antitumour activity of F 11782 relative to etoposide against these early-stage LX-1 tumour xenografts, and this again activity proved more durable since the optimal T/C values for F 11782 were obtained on days 36 and 39, relative to only day 15 with etoposide.

### DISCUSSION

This study has documented major in vivo antitumour activity for this novel lipophilic epipodophylloid, F 11782, against a panel of transplantable murine and human tumour models. F 11782 exhibited marked antitumour activity against the i.v.-implanted murine

© 2000 Cancer Research Campaign

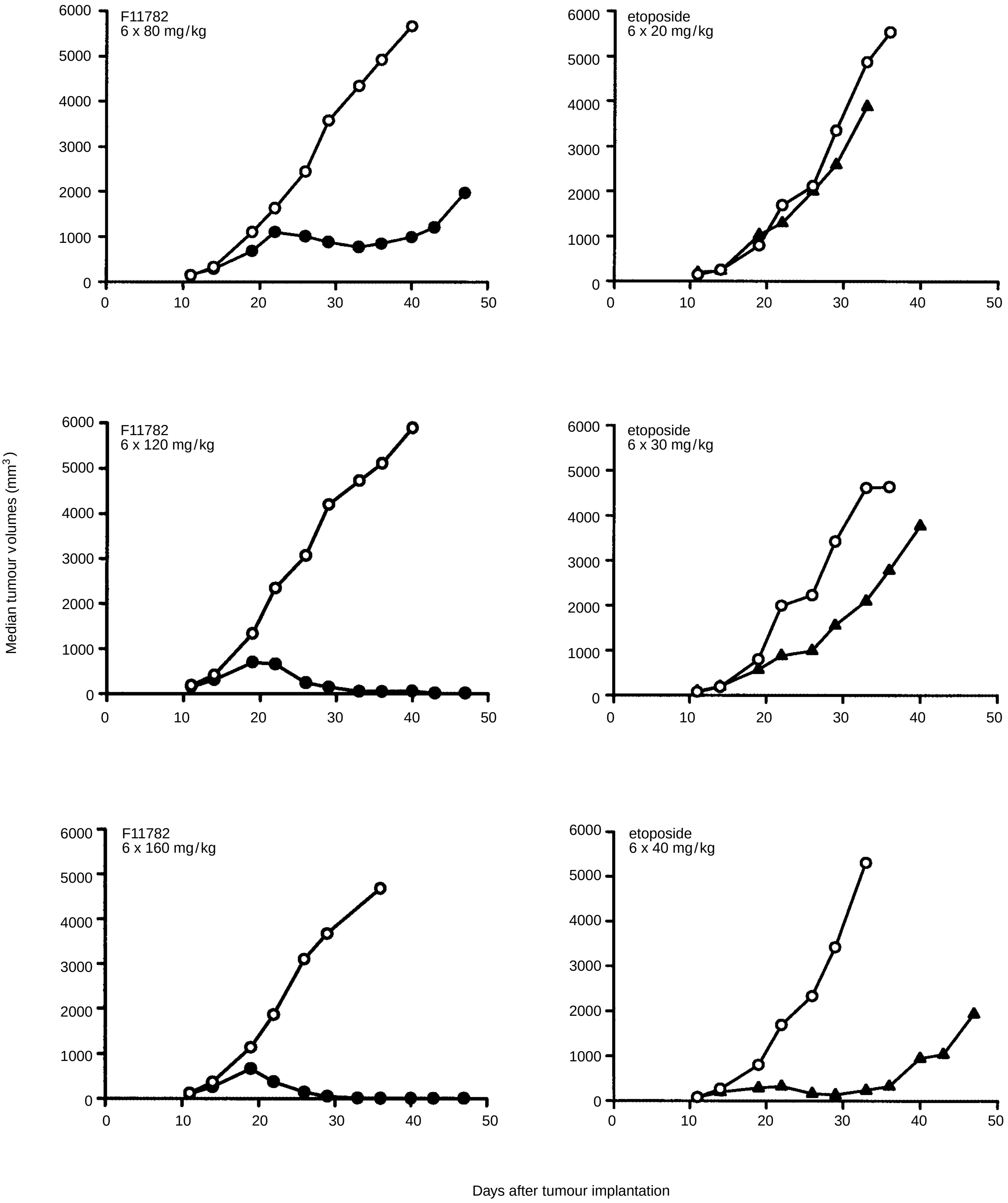

**Figure 2** Responses of human advanced-stage MX-1 (breast) xenografted tumours to 80, 120 or 160 mg/kg per injection F 11782 (●) or 20, 30 and 40 mg/kg per injection etoposide (▲), relative to control vehicle-treated mice (○). Tumour fragments were implanted s.c. by trocar into athymic mice and, when tumours had reached a volume of 10–300 $mm^3$, mice were randomized and treatments were given i.p. on days 11, 13, 15, 18, 20 and 22

*© 2000 Cancer Research Campaign*

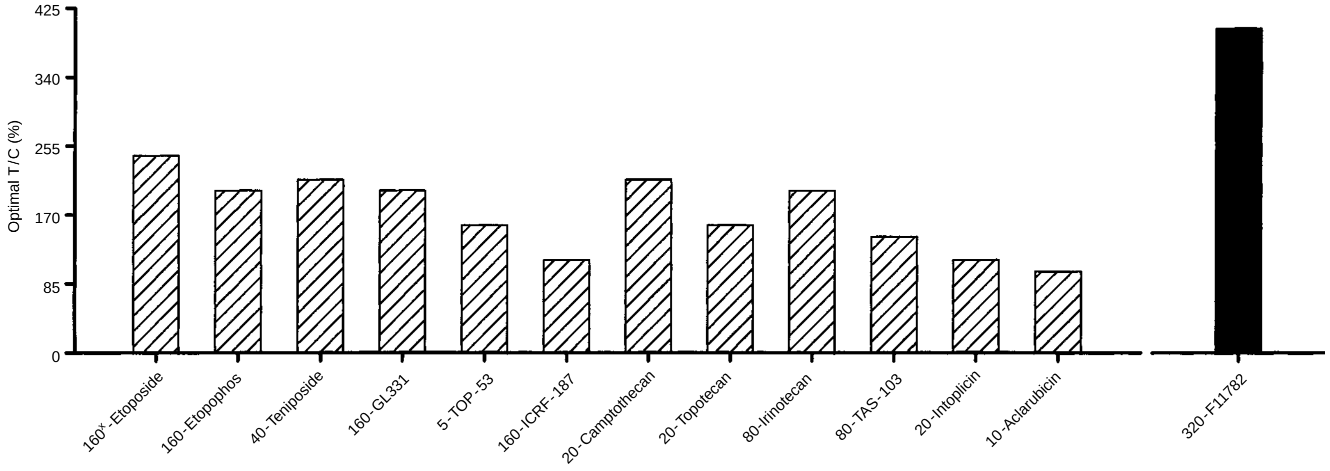

**Figure 3** Effects of F 11782 and a series of 12 topoisomerases poisons or inhibitors, given i.p. as a single dose on day 1 at their optimal doses (x) indicated as mg/kg, on the survival of mice bearing the murine P388 leukaemia grafted i.v.

P388 leukaemia in terms of survival prolongation, when given i.p. as a single treatment over doses ranging from 40–320 mg/kg, yielding $T/C_{ls}$ ratios of 143–400%, with no associated toxicity. The maximal $T/C_{ls}$ value of 400%, indicative of a high level of antileukaemic activity according to NCI criteria (Venditti, 1981), is 1.6 to 1.9-fold superior to those recorded with etoposide and camptothecin, and 1.9 to 4-fold superior to those noted with a series of 10 topoisomerases poisons or inhibitors tested concurrently under the same experimental conditions (see Figure 3, unpublished data).

Intermittent treatments over two weeks permitted administration of the highest total dosage of F 11782, namely 960 mg/kg, corresponding to three times the highest single dose. Antitumour efficacy was recorded over six dose levels of F 11782 using this intermittent schedule, and over four and five dose levels with the single-dose or the four daily injections, respectively. Therefore, intermittent multiple treatments over two weeks appeared the best schedule for F 11782 in this P388 model.

F 11782 also proved active in this model when administered i.v. or p.o. A single i.v. dose of 80–150 mg/kg and multiple p.o. doses (80–320 mg/kg/injection) over two weeks, resulted in a high level of activity. These repeated oral doses of F 11782 had definite antitumour activity over six dose levels, as well as showing that a 6-fold higher total dosage could be given thus, without any adverse side effects. This good overall tolerance to F 11782 suggests that this novel compound may have a wide therapeutic window. These results also indicate that F 11782 readily crosses physiological barriers and is well absorbed by the mouse, auguring well for clinical studies if similar absorption is achieved in man.

However, drug solubility was a limiting factor in the dose escalation of F 11782 and no frankly toxic doses could be identified. Therefore, an alternative formulation of F 11782 is required and is currently being sought.

The s.c.-implanted B16 melanoma was selected on account of its relatively drug-refractory nature, with doxorubicin, a clinically widely-used topoisomerase II poison, being described as inactive (Venditti, 1975). F 11782 exhibited marked and highly significant antitumour activity against this model, both in terms of increased life span and tumour growth inhibition, representative of a high level of activity, according to standard criteria reflected by T/C ratios of < 10% (Bissery et al, 1991). This activity was achieved with a single dose, four daily injections or intermittent treatments over two weeks. Superior activity resulted from the intermittent treatments over two weeks, with four dose levels providing statistically significant results, reaching levels of $P < 0.001$, and tumour growth inhibitions of 76–99.8%. Multiple treatments over two weeks also allowed administration of the highest total dose of 480 mg/kg, 1.5-fold higher than that permitted using the other schedules. F 11782 induced body weight loss in excess of 15% in these B16 tumour-bearing mice at the highest dose tested, without any apparent toxicity over the range of 20–80 mg/kg/injection, indicative of a good overall tolerance to F 11782. This activity was again markedly superior to that noted with etoposide, tested concurrently.

Against the experimental pulmonary B16F10 melanoma metastases model F 11782 induced a marked dose-dependent reduction of B16F10 metastatic foci number in the lungs, indicating that it is capable of potently inhibiting their lung colonization.

In spite of experimental limitations and considerable expense, the nude mouse–human tumour model has become widely integrated into evaluation of and screening for new compounds and therapies, and has proved a valuable model (Fiebig et al, 1992; Langdon et al, 1994). MX-1 breast cancer xenografts were previously shown to be predictive of human response to anticancer drugs (Plowman et al, 1997). The present study has shown that F 11782 induced a major and sustained high level of antitumour activity against advanced-staged MX-1 (breast) tumour xenografts, with T/C ratios < 10% being recorded, and long-lasting definite tumour regressions being recorded. Tumour regressions in animal experimental tumour models are considered an important end-point of clinical relevance (Plowman et al, 1997). These effects were noted without any significant body weight changes, indicating that in these models too treatments with F 11782 were well tolerated. Against early-stage LX-1 (lung) xenografts, F 11782 also showed definite antitumour activity, as reflected by a maximal tumour growth inhibition of 81%, which again was not associated with any significant body weight loss. A direct comparison with etoposide tested concurrently showed clear

© 2000 Cancer Research Campaign superiority for F 11782 against both MX-1 and LX-1 tumour xenografts. Only modest sensitivity to etoposide or etopophos was reported earlier using this LX-1 model (Rose et al, 1990), whilst a lack of activity for etoposide was claimed using a qdx5 schedule and the i.v. route by Utsugi et al (1996). In addition, doxorubicin has been described as inactive, when given as i.p. intermittent doses, in both the MX-1 and LX-1 models (Ovejera and Houchens 1981). More recently, inactivity for doxorubicin against the MX-1 xenografts has also been reported, with an optimal T/C value of 55% resulting from a single i.v. drug administration (Pratesi et al, 1996).

Therefore, the results of this preclinical in vivo study show that F 11782 exhibited marked activity against a panel of experimental tumours with different biological properties and chemosensitivities. This activity was obtained consistently over a wide range of 2–6 dose levels without major toxicity, as judged by monitoring body weight loss or early deaths, demonstrating overall a high level of tolerance to F 11782 and suggesting a high therapeutic index. F 11782 has been selected for preclinical development and Phase I clinical trials are scheduled to commence at the end of 2000. In the meantime detailed pharmacokinetics as well as toxicological studies are underway.

### ACKNOWLEDGEMENTS

We are grateful to Dr AD Degryse for kindly performing the autopsies and providing her expert opinion. We also thank Jacqueline Astruc, Eric Chazottes, Géraldine Berrichon and Caroline Dejean for their skilled technical assistance and Christel Ricome, Olivier Lafosse and Marie Manicci for their invaluable data-processing assistance.

### REFERENCES

Bissery M-C, Guénard D, Guéritte-Voegelein F and Lavelle F (1991) Experimental antitumour activity of taxotere (RP 56976, NSC 628503), a taxol analogue. *Cancer Res* **51**: 4845–4852

Fiebig HH, Berger DP, Dengler WA, Wallbrecher E and Winterhalter BR (1992) Combined *in vitro/in vivo* test procedure with human tumour xenografts for new drug development. In: *Immunodeficient Mice in Oncology*, Fiebig HH and Berger DP (eds), pp 321–351. Karger, Basel

Fortune JM, Velea L, Graves DE, Ustugi T, Yamada Y and Osheroff N (1997) Antitumour activity of a novel quinolone derivative, TAS-103, with inhibitory effects on topoisomerases I and II. *Jpn J Cancer* **88**: 992–1002

Froelich-Ammon SJ and Osheroff N (1995) Topoisomerase Poisons: Harnessing the Dark Side of Enzyme Mechanism *J Biol Chem* **270**: 21429–21439

Gatto B, Capranico G and Palumbo M (1999) Drugs acting on DNA topoisomerases: recent advances and future perspectives. *Curr Pharm Design* **5**: 195–215

Hendriks HR, Langdon S, Berger DP, Breistol K, Fiebig HH, Fodstad Ø and Schwartsmann G (1992) Comparative antitumour activity of vinblastine-isoleucinate and related vinca alkaloids in human tumour xenografts. *Eur J Cancer* **28A**: 767–773

Hussain I, Mohler JL, Seigler HF and Besterman JM (1994) Elevation of topoisomerase I messenger RNA, protein, and catalytic activity in human tumour: demonstration of tumour-type specificity and implications for cancer chemotherapy. *Cancer Res* **54**: 539–546

Ishida R, Hamatake M, Wasserman RA, Nitiss JL, Wang JC and Andoh T (1995) DNA topoisomerase II is the molecular target of bisdioxopiperazine derivatives ICRF-159 and ICRF-193 in *Saccharomyces cerevisia. Cancer Res* **55**: 2299–2303

Khélifa T and Beck WT (1999) Merbarone, a catalytic inhibitor of DNA topoisomerase II, induces apoptosis in CEM cells through activation of ICE/CED-3-like protease. *Mol Pharmacol* **55**: 548–556

Kruczynski A, Colpaert F, Tarayre J-P, Mouillard P, Fahy J and Hill BT (1998) preclinical in vivo antitumour activity of vinflunine, a novel fluorinated *Vinca* alkaloid. *Cancer Chemother Pharmacol* **41**: 437–4478

Langdon SP, Hendriks HR, Pratesi G, Berger DP, Fodstad Ø, Fiebig HH and Boven E (1994) Preclinical phase II studies in human tumour xenografts: a European multicenter follow-up study. *Ann Oncol* **5**: 415–422

Ovejera A and Houchens DP (1981) Human tumour xenografts in athymic nude mice as a preclinical screen for anticancer agents. *Semin Oncol* **8**: 386–393

Perrin D, van Hille B, Barret J-M, Kruczynski A, Etiévant C, Imbert T and Hill BT (2000) F 11782, a novel epipodophylloid non-intercalating dual catalytic inhibitor of topoisomerases I and II with an original mechanism of action. *Biochem Pharmacol* **59**: 807–820

Plowman J, Dykes DJ, Hollingshead M, Simpson-Herren L and Alley MC (1997) Human tumour xenograft models in NCI drug development. In: *Anticancer Drug Development Guide*, Teicher BA (ed), pp 101–125. Humana Press Inc, Totowa, NJ

Pommier Y (1997) DNA topoisomerase II inhibitors. In: *Cancer Therapeutics: Experimental and Clinical Agents*, Teicher BA (ed), pp 153–174. Humana Press Inc, Totowa, NJ

Pratesi G, De Cesare M and Zunino F (1996) Efficacy of lonidamine combined with different DNA-damaging agents in the treatment of the MX-1 tumour xenografts. *Cancer Chemother Pharmacol* **38**: 123–128

Riou J-F, Helissey P, Grondard L and Giorgi-Renault S (1991) Inhibition of eukaryotic DNA topoisomerase I and II activities by indoloquinolinedione derivatives. *Mol Pharmacol* **40**: 699–706

Rose WC, Basler GA, Trail PA, Saulnier M, Crosswell AR and Casazza AM (1990) Preclinical antitumour activity of a soluble etoposide analog, BMY-10481–30. *Invest New Drugs* **8**: 525–532

Utsugi T, Shibata J, Sugimoto Y, Aoyagi K, Wierzba K, Kobunai T, Terada T, Oh-hara T, Tsuruo T and Yamada Y (1996) Antitumour activity of a novel podophyllotoxin derivative (TOP-53) against lung cancer and lung metastatic cancer. *Cancer Res* **56**: 2809–2814

van der Zee AGJ, Hollema H, de Jong S, Boonstra H, Gouw A, Willemse PHB, Zijlstra JG and de Vries EGE (1991) P-glycoprotein expression and DNA topoisomerase I and II activity in benign tumours of the ovary, before and after platinum/cyclophosphamide chemotherapy. *Cancer Res* **51**: 5915–5920

Venditti JM (1975) Relevance of transplantable animal-tumour systems to the selection of new agents for clinical trials. In: *Pharmacological Basis of Cancer Therapy*, Venditti JM (ed), pp 245–270 Williams and Wilkins Company, Baltimore

Venditti JM (1981) Preclinical drug development: rationale and methods. *Semin Oncol* **8**: 349–361

Yamashita Y, Kawada S, Fujii N and Nakano H (1991) Induction of mammalian DNA topoisomerase I and II mediated DNA cleavage by saintopin, a new antitumour agent from fungus. *Biochemistry* **30**: 5838–5845

*© 2000 Cancer Research Campaign*
